# Formation of Phosphoglycosides in *Caenorhabditis elegans*: A Novel Biotransformation Pathway

**DOI:** 10.1371/journal.pone.0046914

**Published:** 2012-10-17

**Authors:** Sebastian T. Soukup, Britta Spanier, Gregor Grünz, Diana Bunzel, Hannelore Daniel, Sabine E. Kulling

**Affiliations:** 1 Department of Safety and Quality of Fruit and Vegetables, Federal Research Institute of Nutrition and Food, Max Rubner-Institut, Karlsruhe, Germany; 2 ZIEL Research Center for Nutrition and Food Sciences, Department of Biochemistry, Technische Universität München, Freising, Germany; Concordia University Wisconsin, United States of America

## Abstract

**Background:**

*Caenorhabditis elegans (C. elegans)* has become a widely used model to explore the effect of food constituents on health as well as on life-span extension. The results imply that besides essential nutrients several flavonoids are able to impact the aging process. What is less investigated is the bioavailability and biotransformation of these compounds in *C. elegans*. In the present study, we focused on the soy isoflavone genistein and its metabolism in the nematode as a basis for assessing whether this model system mimics the mammalian condition.

**Principal Findings:**

*C. elegans* was exposed to 100 µM genistein for 48 hours. The worm homogenate was extracted and analyzed by liquid chromatography (LC). 11 metabolites of genistein were detected and characterized using LC electrospray ionization mass spectrometry. All genistein metabolites formed by *C. elegans* were found to be sugar conjugates, primarily genistein-*O*-glucosides. The dominant metabolite was identified as genistein-7-*O*-phosphoglucoside. Further interesting metabolites include two genistein-di-*O*-glycosides, a genistein-*O*-disaccharide as well as a genistein-*O*-phosphodisaccharide.

**Conclusions/Significance:**

Our study provides evidence for a novel biotransformation pathway in *C. elegans* leading to conjugative metabolites which are not known for mammals. The metabolism of genistein in mammals and in *C. elegans* differs widely which may greatly impact the bioactivity. These differences need to be appropriately taken into consideration when *C. elegans* is used as a model to assess possible health or aging effects.

## Introduction

Metabolic transformation plays a key role in mammalian and non-mammalian defense against xenobiotics, which might exert beneficial or harmful effects on biological systems. Biotransformation generally includes phase I reactions in which functional groups are either introduced to the xenobiotic or exposed, followed by conjugation reactions with water-soluble moieties. These chemical modifications change the physicochemical properties of the xenobiotic which in turn alters also its biological and physiological effects. Consequently, detailed information on the metabolism of a foreign compound is crucial for a reliable assessment of potential benefits but also risks associated with its exposure. When using an animal model to investigate the biological activity of a substance it is important to keep in mind that its response, especially its metabolism, may differ between the model system and that of humans.

Non-mammalian model organisms such as the nematode *Caenorhabditis elegans* (*C. elegans*) have become an important tool in systems biology research. The genome of *C. elegans* is fully sequenced and many physiological functions found in mammals have been identified in the nematode as well. Due to the relatively high degree of complexity and the small total number of 959 somatic cells *C. elegans* provides a controllable experimental system employed in developmental research, cell physiology and aging [1].

The aging trajectory is controlled by genetic mechanisms involving signal transduction pathways that are evolutionarily conserved in yeasts, worms, flies and mammals including humans [2]. In nutrition research, *C. elegans* has become a widely used model to explore the effect of food constituents on health as well as on life-span extension. The results imply that essential nutrients like tocopherols but also plant secondary compounds from fruit, vegetables and herbs, especially polyphenols, are able to impact the aging process. Such effects on life span of *C. elegans* have recently been described for example for the proanthocyanidin fraction derived from blueberries, epigallocatechin gallate from green tea, or quercetin derivatives from onions [3] [4] [5]. However, what is frequently not assessed in those studies is the extent by which the test compounds are bioavailable and moreover, to which extent and into which metabolites the food-derived compounds are converted. Knowledge about metabolic conjugation reactions in *C. elegans* is thus very limited and so far, only a few reports have addressed this question [6] [7].

We recently assessed for four structurally related flavonoids the apparent bioavailability in *C. elegans* and observed indications for a substantial metabolism [8]. In the present study we characterized the biotransformation of genistein, a prominent isoflavone found in soy and soy products by *C. elegans*. Very recently Fischer et al. reported that genistein affect the immunity of the worm by acting antiestrogenic via a reduced expression of the estrogen-responsive vitellogenin-genes [9].

In mammalian systems the metabolism of genistein is well described and reveals an impressive complexity [10]. Our aim was to identify and quantify the metabolites to which genistein is converted in *C. elegans* as a basis for assessing whether this model system mimics the mammalian condition.

## Materials and Methods

### Chemicals and reagents

Genistein was purchased from Sigma Aldrich (Steinheim, Germany) with a purity >99.0% as determined by LC/photodiode array detection (DAD). Genistin (genistein-7-*O*-β-d-glucoside) was bought from Plantech UK (Reading, UK), sophoricoside (genistein-4′-*O*-β-d-glucoside) was from ChromaDex (Irvine, USA) and genistein-4′,7-di-*O*- β-D-glucoside was purchased from Apin Chemicals Ltd. (Abingdon, UK). Maltodextrin DE 4.0–7.0 was purchased from Sigma Aldrich (Steinheim, Germany) and cyclodextrin glucanotransferase was provided by Amano Enzyme Europe Ltd. (Oxfordshire, UK). All solvents used were of analytical grade. A stock solution of genistein was prepared using ethanol∶Tween 80 (Sigma Aldrich, Steinheim, Germany) (92∶8, by volume) as a solvent.

### Enzymatic formation of genistein-7-*O*-β-d-maltoside and genistein-4′-*O*-β-d-maltoside

Genistein-7-*O*-β-d-maltoside and genistein-4′-*O*-β-d-maltoside were enzymatically prepared by incubation of genistein-7-*O*-β-d-glucoside and genistein-4′-*O*-β-d-glucoside, respectively, with maltodextrin and cyclodextrin glucanotransferase as described [11].

### *Caenorhabditis elegans* strain and culture conditions

*C. elegans* (strain Bristol N2) and *Escherichia coli* (*E. coli*) strain OP50 were obtained from the Caenorhabditis Genetics Center (CGC), University of Minnesota, USA. The nematodes were grown at 20°C on nematode growth medium (NGM) agar plates as previously described [12] [13]. We used heat killed *E. coli* OP50 as food source in order to prevent any bacterial transformation of genistein. Bacteria were grown overnight, concentrated 5-fold by centrifugation and heat killed at 65°C for 30 min according to [14]. Heat killed OP50 feeding solution and genistein stock solution were added to the NGM plates. The final concentration of genistein was 100 µM.

### Sample preparation

The nematodes were grown on NGM plates containing 100 µM of genistein for 48 hours. After exposure, worms were washed five times with 0.1% bovine serum albumin (w/v) to remove all adhering genistein. The washed worm pellets were frozen in liquid nitrogen until analysis. For analysis, worm samples were thawed and treated with a FastPrep homogenizer (4×15 sec) using 1-mm silica spheres with intermediate cooling periods on ice (FastPrep-24, MP Biomedicals, Solon, USA). A 50 µl aliquot of the homogenate was mixed with 150 µl of methanol and the suspension was centrifuged (10 min, 16,000× g, 4°C). An aliquot of the supernatant was subsequently diluted with water (1∶1) and analyzed by LC-DAD and LC-MS.

### LC-DAD and LC-MS analyses

The LC-DAD analyses were performed on a Shimadzu LC system equipped with a controller (CBM-20A), a degasser (DGU-20A3), two pumps (LC-20AD), an autosampler (SIL-20AC HT), a column oven (CTO-20AC) and a DAD (SPD-M20A). The LC system was controlled by the software LCsolution 1.24. LC separation was carried out on a YMC Pack Hydrosphere C18 column (150×3.0 mm, particle size 3 µm) equipped with a Phenomenex SecurityGuard (C18, 4.0×3.0 mm). Eluent A was ammonium acetate (25 mM, pH 7.0) and eluent B was acetonitrile. A linear gradient was used with a flow rate of 0.8 ml/min and the following elution profile: 0–5 min isocratic with 10% B, 5–16 min from 10% to 20% B, 16–30 min from 20% to 42% B, 30–31 min from 42% to 95% B, 31–36 min isocratic with 95% B, 36–37 min from 95% to 10% B and 37–47 min isocratic with initial conditions. The column oven and the DAD flow cell were adjusted to 40°C. The injection volume was 20 µl. The chromatograms were recorded from 200 to 400 nm and the 260 nm trace was used to monitor the analytes.

The LC-MS analyses were performed on two systems. The first system was an ABSciex QTrap 5500 mass spectrometer equipped with a Shimadzu LC system, which consisted of a controller (CBM-20A), a degasser (DGU-20A5), two pumps (LC-30AD), an autosampler (SIL-30AC) and a column oven (CTO-20AC). The LC-MS system was controlled by the software Analyst 1.5.2. The LC conditions were the same as described above. The electrospray ionization (ESI) source was operated in the negative mode using the following parameters: Curtain gas (CUR) 20 psi, ion spray voltage (IS) −3500 V, ion source gas-1 (GS 1) 60 psi, ion source gas-2 (GS 2) 40 psi, ion source gas-2 temperature (TEM) 600°C. The MS parameters were adjusted as follows: scan rate 20,000 Da/sec, declustering potential (DP) −250 V, entrance potential (EP) −10 V. The MS full scans (enhanced MS mode) were performed with a scan range from 100 to 755 m/z and a collision energy voltage (CE) of −10 V. The MS/MS measurements (enhanced product ion mode) were executed with a collision energy voltage (CE) of −40 V and collision energy spread of 30 V. Nitrogen was used as collision gas.

The second LC-MS system was an Agilent 6540 QToF mass spectrometer equipped with an Agilent 1290 Infinity LC system, which consisted of a controller, a degasser, a binary pump, an autosampler, a column oven and a DAD. The system was controlled by the software MassHunter B.03.01 Build 3.1.346.0. The LC conditions were the same as described above. The jet stream ESI source was operated in the negative mode and the MS parameters were adjusted as follows: Nebulising pressure 50 psi, gas temperature 350°C, gas flow 10 l/min, capillary voltage (VCap) 3500 V, skimmer voltage 65 V, fragmentor voltage 175 V, Oct 1 RF Vpp voltage 750 V, scan range from 100 to 1100 m/z, scan rate 1 spectrum/sec. The MS/MS experiments were performed with a CID voltage of 30 V. Nitrogen was used as collision gas.

## Results

**Figure 1:**
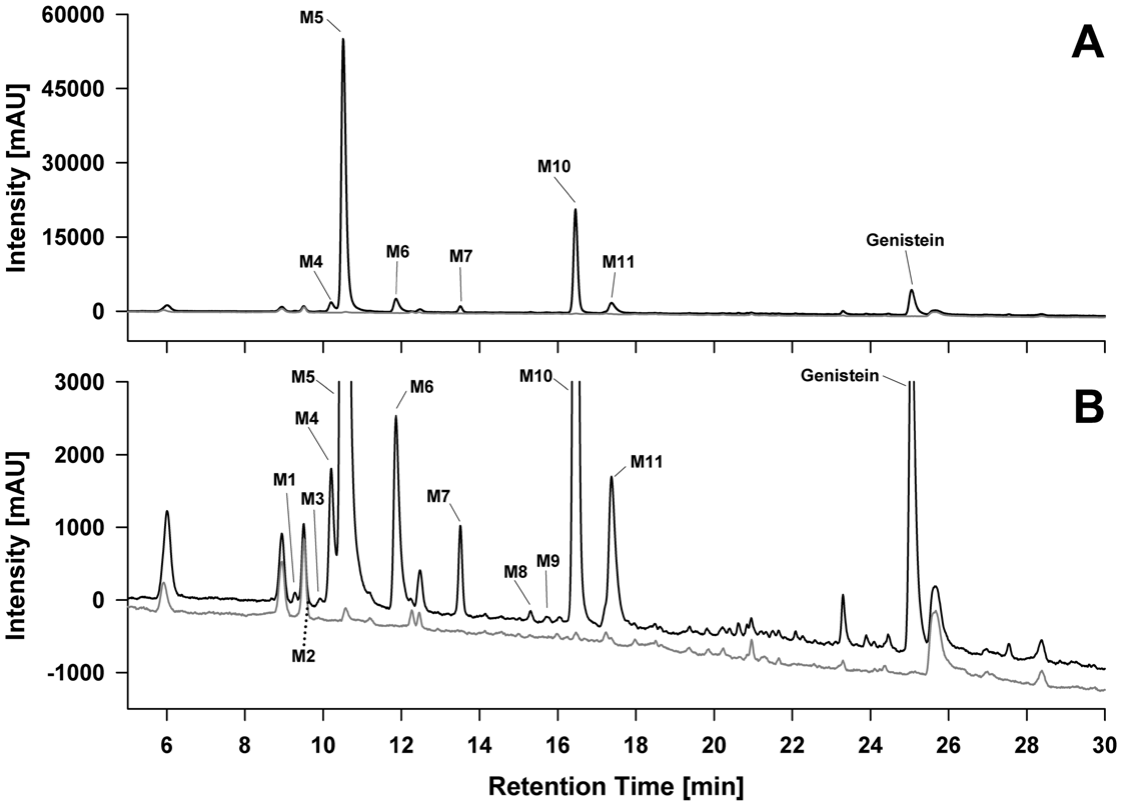
Representative LC-UV chromatogram of the organic *C. elegans* extract after exposure of nematodes to genistein. Incubations were performed with genistein (black line) and without genistein (controls, grey line) for 48 hours. The chromatogram was monitored at 260 nm and is displayed on two different scales: (A) Full scale chromatogram showing the dominance of M5 and M10, which represent around 80% of the genistein metabolites based on the peak areas. (B) Zoomed chromatogram showing the detailed genistein metabolite spectrum. M2 (marked by a dashed line) was too small to be visualized using UV detection but was detected by mass spectrometry.

*C. elegans* converted the isoflavone genistein to at least 11 metabolites which were detected and characterized by LC-DAD and LC-MS analysis. **Figure 1** shows the LC-UV chromatogram of the organic extract derived from *C. elegans* exposed to 100 µM genistein for 48 h. The peaks (denoted as M1 to M11) were numbered according to their order of elution. All peaks that were not present in control incubations without genistein were assumed to be metabolites and characterized by interpretation of their MS, MS/MS and MS-TOF data. In sum, all metabolites detected were identified as genistein sugar conjugates. In the following, the mass spectrometric data of each metabolite will be discussed and explained.

### Detection of genistein-*O*-monohexosides

**Figure 2:**
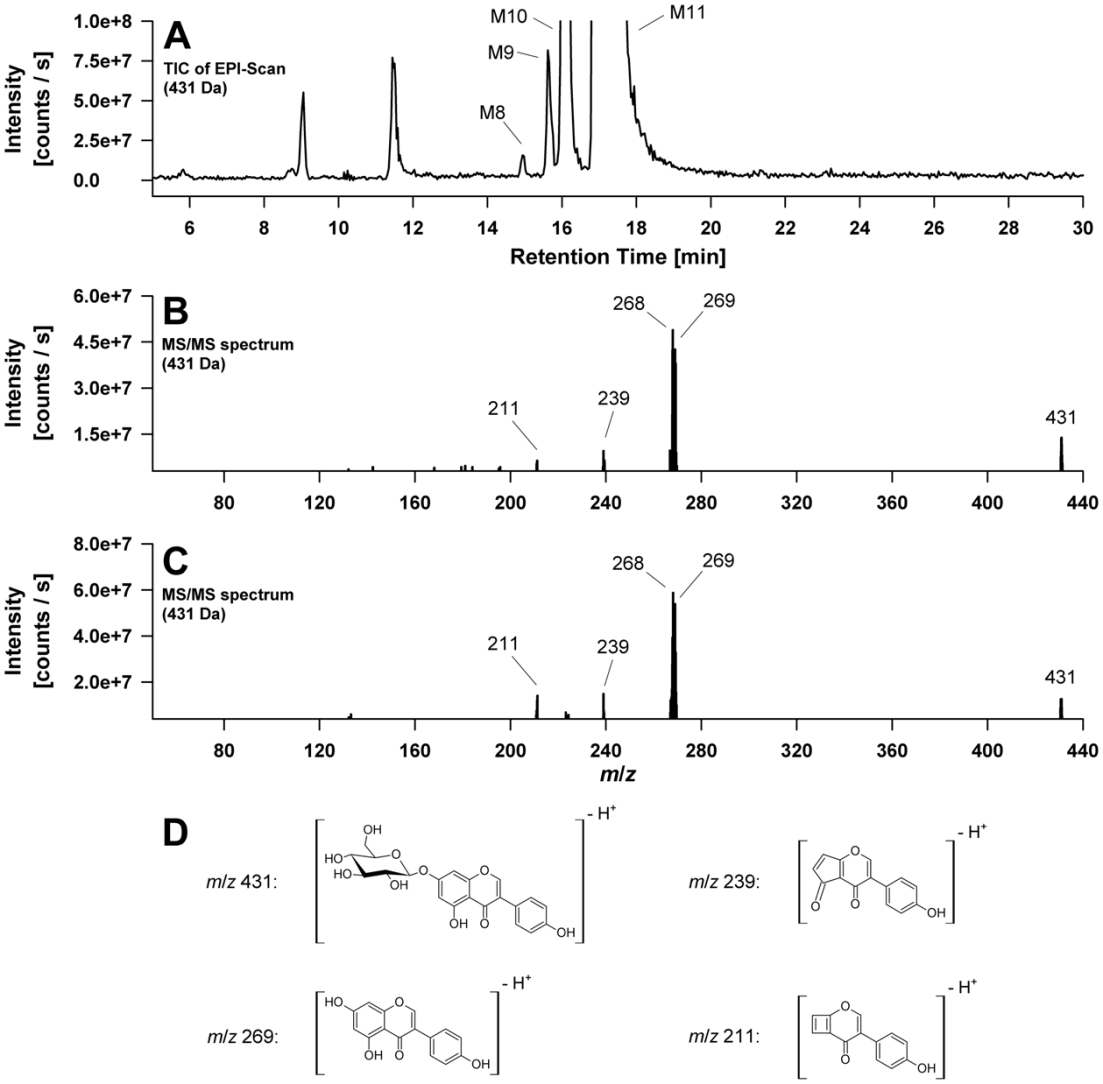
Electrospray mass spectrometric detection (QTrap system) of genistein-*O*-monohexosides formed by *C. elegans*. (A) Total ion chromatogram (TIC) of the enhanced product ion (EPI) scan of the *m/z* 431 precursor showing four genistein-*O*-monohexoside metabolites. The two major ones are named M10 and M11; two additional minor metabolites are marked as M8 and M9. (B) MS/MS spectrum of the standard compound genistein-7-*O*-ß-D-glucoside. (C) MS/MS spectrum of metabolite M10, identified as genistein-7-*O*-ß-D-glucoside using the corresponding standard compound. (D) Proposed structures of the fragment ions observed in the MS/MS spectra shown under (B) and (C).

Metabolite **10** and **11** were identified as genistein-7-*O*-β-D-glucoside and genistein-4′-*O*-β-D-glucoside, respectively using reference standard compounds. Both MS spectra measured in the negative mode exhibit a high intensity ion at m/z 431 assigned to the [M-H]^−^ ion. The signals at *m/z* 269 and *m/z* 268 correspond to the genistein aglycone fragment in form of the anion (Y^−^) and the radical anion (Y-H)^•−^ respectively, indicating the loss of a glucose moiety via heterolytic (−162 Da) or homolytic (−163 Da) cleavage of the glycosidic bond [15]. The MS/MS spectra of the standard compound genistein-7-*O*-β-D-glucoside and of metabolite M10 are shown in **Figure 2B–D**.

Two minor metabolites defined as peak **8** and **9** additionally exhibited [M-H]^−^ ions at *m/z* 431 indicating two further *O*-glycosylated genistein metabolites comprising a hexose moiety. Because of the similarity of their mass spectra to those of M10 and M11, we assume that these metabolites are genistein-7- and genistein-4′-*O*-hexoside stereoisomers, carrying a different hexose sugar moiety, e.g. galactose instead of glucose. The presence of a genistein-5-*O*-glucoside seems less likely because the 5-OH group of the A-ring is involved in intramolecular hydrogen bond formation with the 4-carbonyl group of the isoflavone C-ring [16]. However, this alternative cannot be completely ruled out. **Figure 2A** illustrates the enhanced product ion (EPI) scan of the *m/z* 431 precursor with the four genistein-*O*-monohexoside metabolites M8, M9, M10, and M11.

### Genistein-di-*O*-hexosides and genistein-*O*-disaccharides

**Figure 3:**
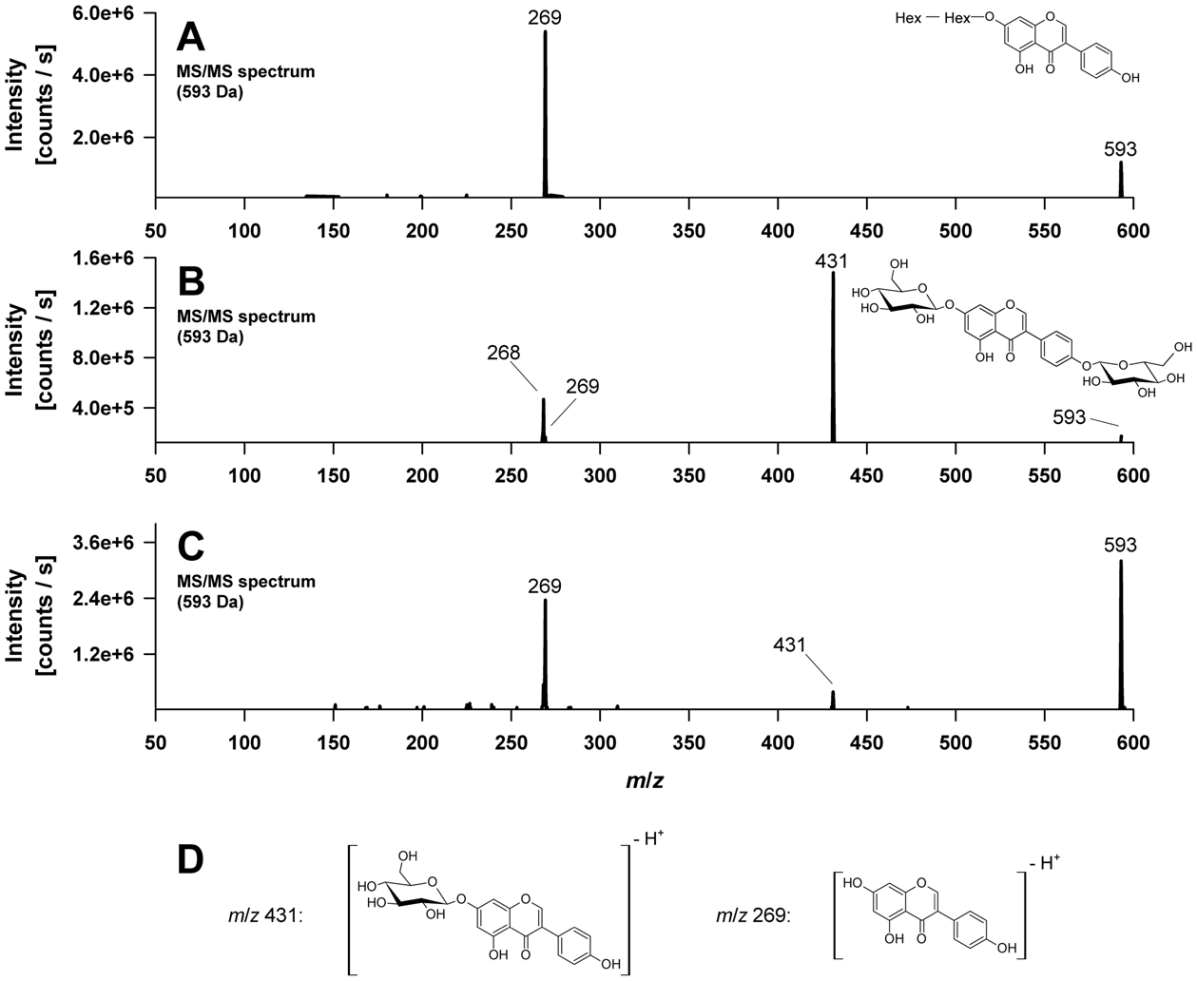
Electrospray mass spectrometric detection (QTrap system) of genistein-di-*O*-hexosides and genistein-*O*-disaccharides formed by *C. elegans*. (A) MS/MS spectrum of metabolite M7, assigned to a genistein-disaccharide with a proposed chemical structure. (B) MS/MS spectrum of metabolite M1, identified as genistein-4′,7-di-*O*-ß-D-glucoside using a standard compound. (C) MS/MS spectrum of metabolite M3, characterized as a genistein-di-*O*-hexoside. (D) Proposed structures of the product ions observed in the MS/MS spectra shown under (A), (B) and (C). Hex, Hexose.

Metabolites **1** and **3** appeared with low abundance. Both produced a molecular [M-H]^−^ ion at *m/z* 593 indicating a genistein molecule plus two monosaccharide units. In both cases the molecular ion was confirmed by precursor ion scans for *m/z* 431, a fragment ion in both peak mass spectra. The detected ions were *m/z* 593 proving the [M-H]^−^ ion, *m/z* 653 for the corresponding solvent adduct [M+CH_3_COO]^−^ ion as well as *m/z* 629 and 631 for the respective chloride ion adducts [M+ ^35^Cl]^−^ and [M+ ^37^Cl]^−^. Besides the *m/z* 431 ion, the product ion mass spectra of *m/z* 593 further show the characteristic genistein aglycone ions at *m/z* 269 and *m/z* 268 (**Figure 3B and C**). Metabolite **1** could be identified as genistein-7,4′-di-*O*-β-D-glucoside by comparison of the product ion mass spectrum with those of an authentic reference compound. The second genistein-di-*O*-hexoside was so far not further characterized.

Metabolite **7** also shows a molecular [M-H]^−^ ion at *m/z* 593 as well as the corresponding chloride ion adducts [M+ ^35^Cl]^−^ and [M+ ^37^Cl]^−^ at *m/z* 629 and *m/z* 631, respectively. However, in contrast to metabolites 1 and 3, assigned as genistein-di-*O*-hexosides, the MS/MS spectrum of peak 7 exhibited, if at all, a relatively low abundance of the fragment ion at *m/z* 431. Furthermore an intense genistein aglycone ion at *m/z* 269 was detected, indicating the neutral loss of 324 Da (*m/z* 593→269), which corresponds to the characteristic fragment mass of an anhydro-disaccharide unit, is preferred (**Figure 3A**). We observed the same behaviour when using *p*-nitrophenyl-β-d-maltoside to study the fragmentation of *O*-disaccharide-substituted phenols. Therefore, genistein-7-*O*-β-d-maltoside and genistein-4′-*O*-β-d-maltoside were prepared enzymatically from genistein-7-*O*-β-d-glucoside and genistein-4′-*O*-β-d-glucoside, respectively. The maltosides had exactly the same mass spectra as peak 7, but eluted later. In conclusion, M7 is a genistein-*O*-disaccharide, conceivably a genistein-glucosyl-glucoside since glucose conjugates represented the vast majority of genistein metabolites formed by *C. elegans*. Because we can exclude the (1→4)-linked maltoside, we propose that M7 most likely is a genistein-*O*-β-glucosyl-(1→6)-glucoside (genistein-gentiobioside) or a genistein-*O*-β-glucosyl-(1→2)-glucoside as these two linkages are the most common ones found in nature. The fragmentation pattern of genistein-di-*O*-hexosides compared to genistein-*O*-disaccharides is depicted in **Figure 3**.

### Detection of genistein-*O*-phospoglucosides

**Figure 4:**
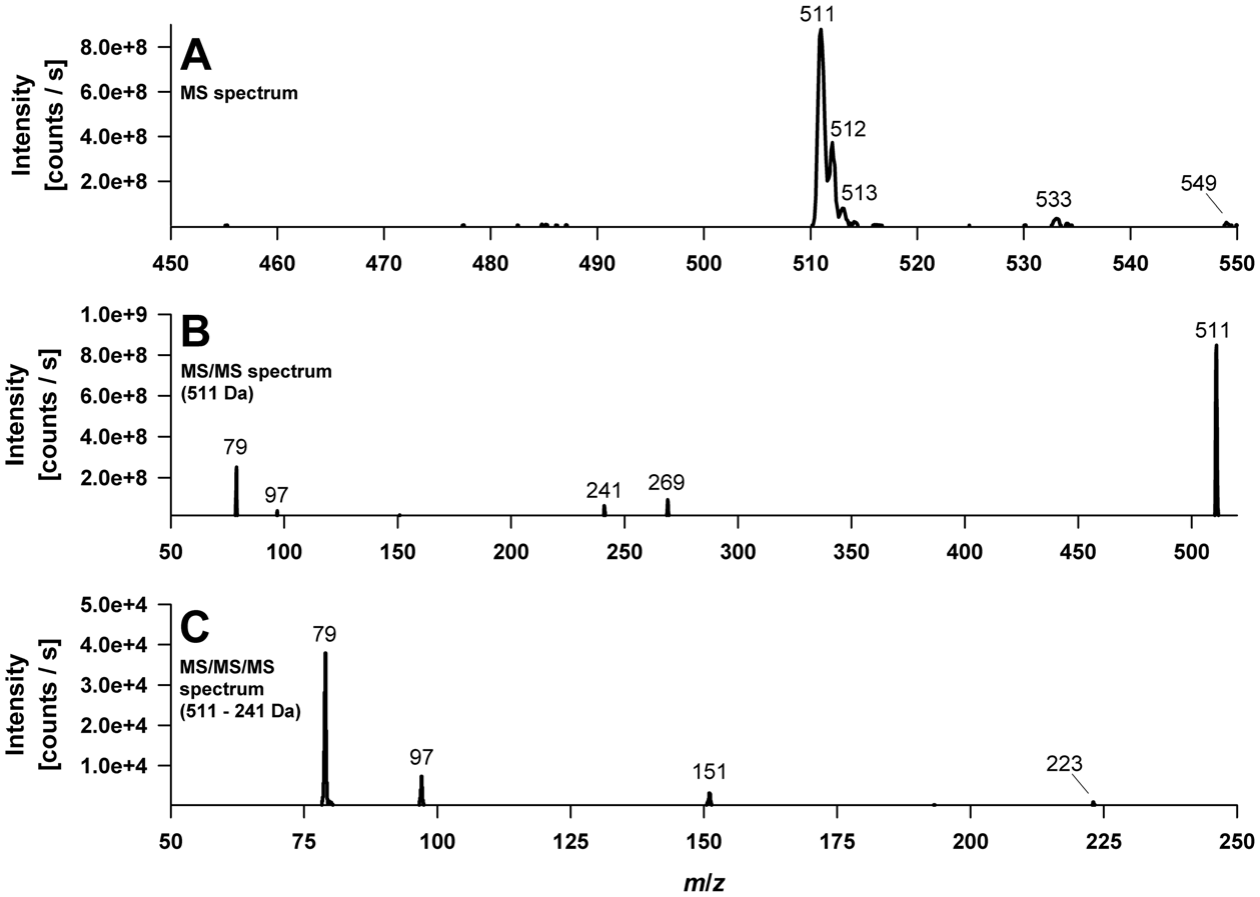
Electrospray mass spectra (QTrap system) of the deprotonated main genistein metabolite M5, identified as a genistein-monophosphoglucoside. (A) MS spectrum. (B) MS/MS spectrum of *m/z* 511. (C) MS/MS/MS spectrum of *m/z* 511→*m/z* 241.

**Figure 5:**
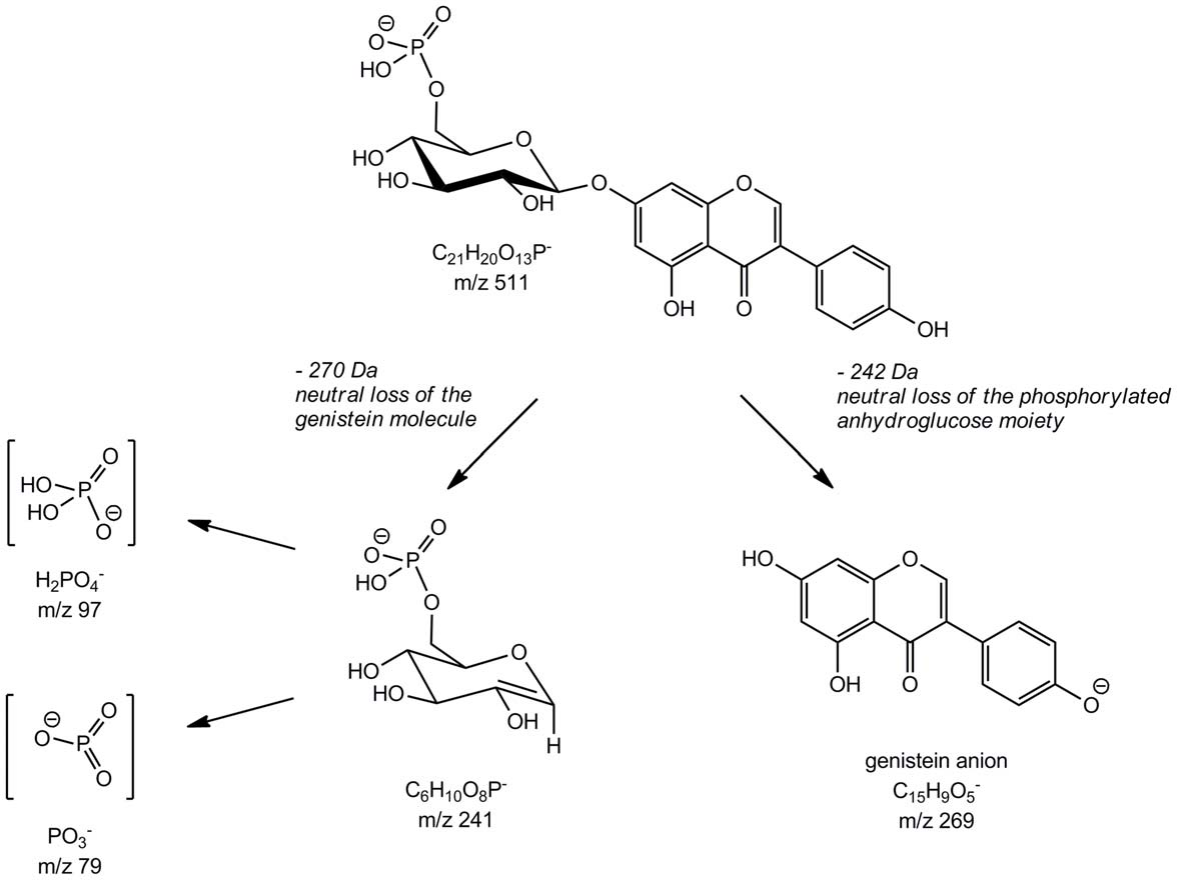
Fragmentation pathway of the main metabolite M5 assumed as genistein-7-*O*-(6″-*O*-phospho)-ß-D-glucoside.

The main metabolite detected was **M5** with a molecular [M-H]^−^ ion at *m/z* 511, which was confirmed by precursor ion experiments scanning for *m/z* 269, the genistein anion. In addition, the corresponding sodium [M-2H+Na]^−^ and potassium [M-2H+K]^−^ adduct ions at *m/z* 533 and *m/z* 549, respectively, were observed (**Figure 4A**). The accurate mass of the molecular ion was determined to be 511.0652 by (ESI)-TOF experiments (**Table 1**). The EPI scan at *m/z* 511 produced, as illustrated in **Figure 4B**, fragment ions at *m/z* 269, 241, 97, and 79. The accurate mass fragment ions were found to be 269.0457, 241.0126, 96.9704, and 78.9593, respectively (Table 1). Based on those accurate mass measurements we conclude that *m/z* 96.9704 and *m/z* 78.9593 represent the negatively charged phosphorous containing fragment ions [PO_3_]^−^ and [H_2_PO_4_]^−^, respectively, deriving from a phosphate monoester. The difference between experimental and theoretical mass was −0.2 mDa for [PO_3_]^−^ and −0.8 mDa for [H_2_PO_4_]^−^, showing a satisfactory agreement. The fragment ion at *m/z* 269.0457 corresponds to the genistein anion resulting from glycosidic bond cleavage and the loss of the uncharged hexose monophosphate moiety C_6_H_11_O_8_P (−242 Da), whereas the ion at *m/z* 241.0126 arose vice versa by the neutral loss of a genistein molecule (−270 Da). The ion peak at *m/z* 241 further indicates that the phosphate group is linked to the hexose moiety (e.g. in *O*-6 position of the hexose) since the MS/MS/MS spectrum of *m/z* 241 showed product ions at *m/z* 79 [PO_3_]^−^ and *m/z* 97 [H_2_PO_4_]^−^ (**Figure 4C**). Overall the MS data indicates that the main metabolite of genistein in *C. elegans* is a genistein-*O*-monophosphohexoside. To confirm these results, peak 5 was isolated and incubated with acid phosphatase, which led to the release of genistein-7-*O*-β-D-glucoside (data not shown). We therefore conclude that the main metabolite formed is a genistein-7-*O*-(6″-*O*-phospho)-β-D-glucoside. The proposed fragmentation pathway is depicted in **Figure 5**.

**Table 1:** Predicted formula, determined and theoretical mass/charge ratios as well as the mass error of fragment ions observed in the product ion mass spectrum of *m/z* 511.

Predicted formula	Determined mass (Da)	Theoretical mass (Da)	Error in mDa (in ppm)
C_21_ H_20_ O_13_ P	511.0652	511.0647	−0.48 (−0.93)
C_15_ H_9_ O_5_	269.0457	269.0455	−0.16 (−0.58)
C_6_ H_10_ O_8_ P	241.0126	241.0119	−0.68 (−2.80)
[H_2_PO_4_]^−^	96.9704	96.9696	−0.82 (−8.51)
[PO_3_]^−^	78.9593	78.9591	−0.22 (−2.77)

Metabolite **6** and **2** occurred in minor amounts and exhibited MS and MS/MS spectra analog to those of M5. Treatment of M6 with acid phosphatase yielded in genistein-4′-*O*-β-D-glucoside suggesting peak M6 to be genistein-4′-*O*-(6″-*O*-phospho)-β-D-glucoside. The third genistein-*O*-monophosho-hexoside M2 was so far not further characterized being only present in trace amounts.

**Figure 6:**
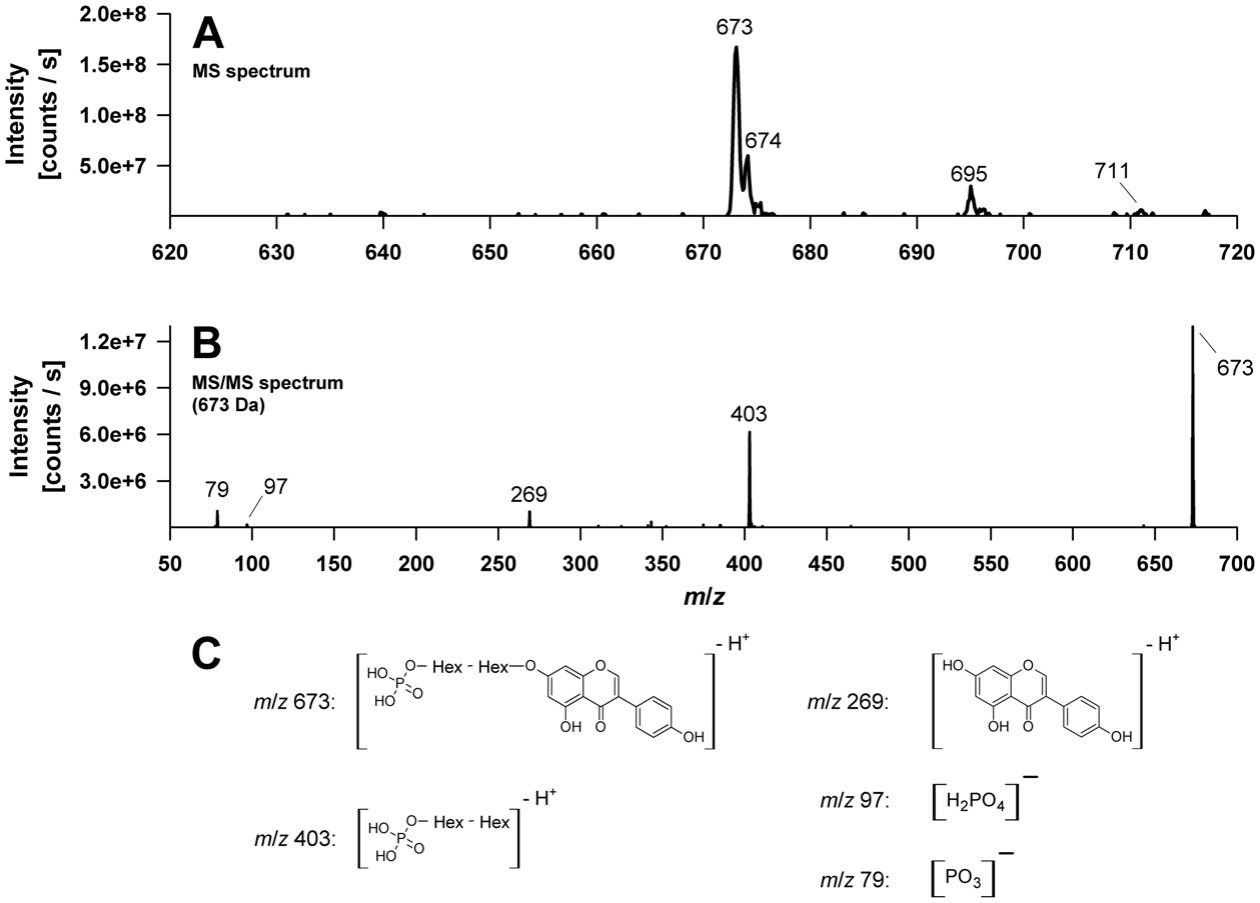
Electrospray mass spectra (QTrap system) of the deprotonated genistein metabolite M4 assigned as a genistein-phosphodisaccharide. (A) MS spectrum. (B) MS/MS spectrum of *m/z* 673. (C) Proposed structures of the product ions observed in the MS/MS spectra. Hex, Hexose.

With *m/z* 673 peak **M4** showed the highest molecular ion of all metabolites detected. This was confirmed by precursor ion experiments and detection of the corresponding sodium [M-2H+Na]^−^ and potassium [M-2H+K]^−^ adduct ions at *m/z* 695 and *m/z* 711, respectively (**Figure 6A**). Fragment ions at *m/z* 79, *m/z* 97 and *m/z* 269 once again suggest the presence of a phosphate group and the genistein aglycone. The occurrence of a fragment ion at *m/z* 269 indicates a genistein anion formed by glycosidic bond cleavage and the loss of a phosphorylated disaccharide unit C_12_H_21_O_13_P (−404 Da), whereas the ion at *m/z* 403 results from a neutral loss of genistein (−270 Da) (**Figure 6B**). Thus we assume that the metabolite M4 is a genistein-*O*-phosphodissacharide.

**Table 2** summarizes the experimental data as well as the proposed structure of each metabolite.

**Table 2:** Experimental data and proposed structures of the metabolites detected after exposure of *C. elegans* to genistein for 48 h.

Peak	Peak area (%)	[M-H]^−^	Fragment ions	Proposed structure
1	0.1	593	431, 269, 268	Gen-7,4′-di-*O*-glucoside
2	0*	511	269, 241, 97, 79	Gen-*O*-phosphohexoside
3	0.1	593	431, 269, 239, 227	Gen-di-*O*-hexoside
4	2.3	673	403, 269, 97, 79	Gen-*O*-phosphodisaccharide
5	61.1	511	269, 241, 97, 79	Gen-7-*O*-phosphoglucoside
6	3.6	511	413, 269, 241, 97, 79	Gen-4′-*O*-phosphohexoside
7	1.0	593	269, 225	Gen-*O*-disaccharide
8	0.1	431	311, 269/268, 225,157/159	Gen-*O*-hexoside
9	0.1	431	311, 269, 225,157/159	Gen-*O*-hexoside
10	19.6	431	269/268, 239, 211, 195, 167	Gen-7-*O*-ß-D-glucoside
11	3.4	431	269/268, 239, 211, 195, 167	Gen-4′-*O*-ß-D-glucoside
	7.3	269	225, 159, 133, 107	Gen aglycone

Peak number and peak area (in %) refer to the LC/UV chromatogram shown in Figure 1.

* Metabolite was only detected in MS analysis. Gen, genistein.

## Discussion

Our studies on the metabolism of the isoflavone genistein in *C. elegans* revealed a novel biotransformation pathway. All metabolites identified are products formed by the worm since the animals were provided with heat-killed bacteria. Moreover, vital *E. coli* were found as unable to produce any of the genistein metabolites detected (data not shown).

All genistein metabolites formed by *C. elegans* were identified as sugar conjugates, primarily genistein-*O*-glucosides. Similar glucosylation reactions of flavonoids in non-mammalian systems are primarily reported for some bacteria species, especially aerobic or facultative anaerobic rod-shaped bacteria such as *Bacillus subtilis*, *Bacillus cereus*, *Xanthomonas campestris* and *Lactobacillus delbrueckii* [17] [18]. More recently Laing et al. [6] were the first to describe an analogous conjugation reaction in the nematode *C. elegans* where a glucose moiety was introduced into the anthelmintic drug albendazole as a xenobiotic response and a variety of quercetin glycosides including sulfate-glycosyl derivatives were recently found by Surco-Laos et al. [7] as metabolites of the flavonoid quercetin by *C. elegans*.

**Figure 7:**
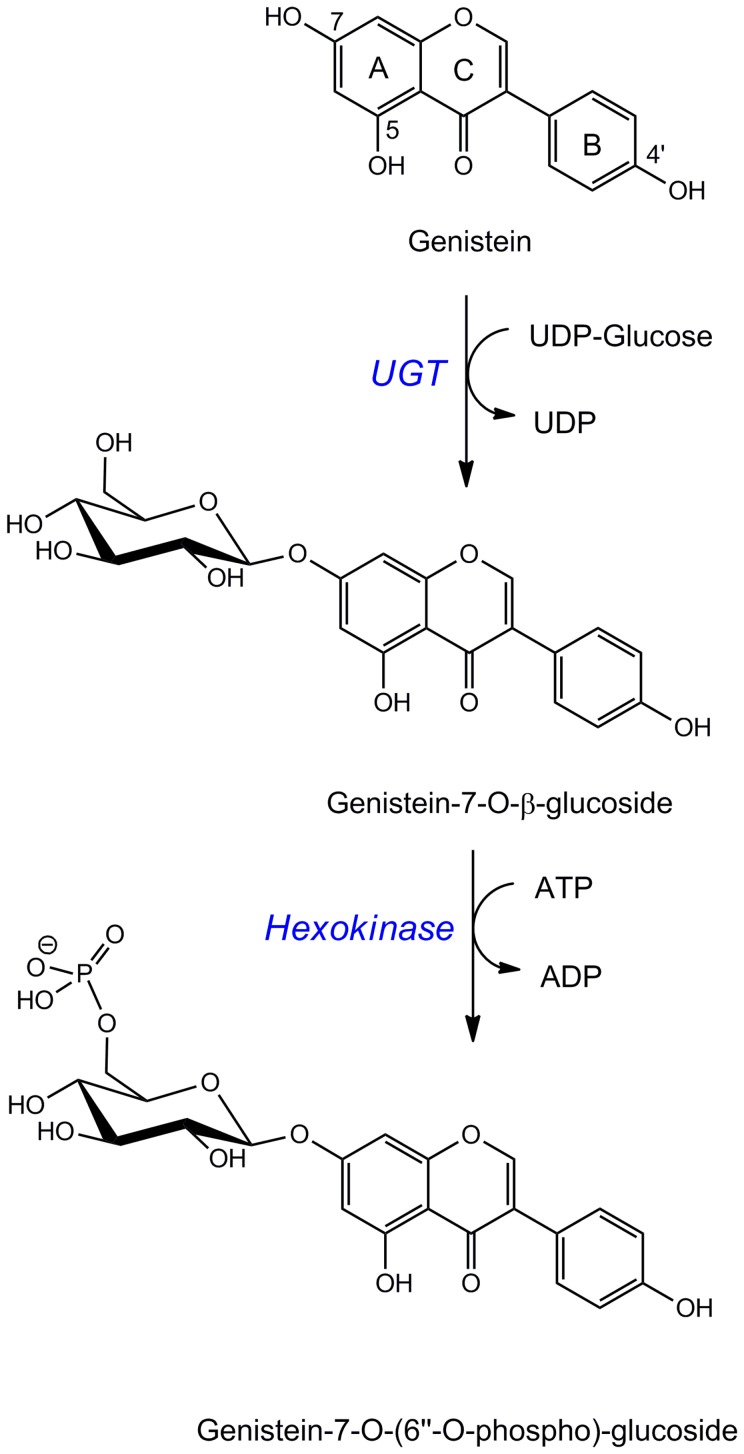
Proposed pathway for the conjugative metabolism of *C. elegans* leading to the main metabolites. UGT, UDP-glycosyltransferase.

In our study, the dominant genistein metabolite in N2 *C. elegans* was reliably identified as a 7-*O*-phosphoglucoside. To our knowledge, this is the first time that phosphoglucosides as biotransformation reaction products are identified in *C. elegans*. By the high concentrations found, it seems that this is the predominant conjugation pathway in the worms. Since we observed both, the genistein-7-*O*-glucoside and the corresponding phosphoglucoside, we speculate that the formation may occur in a two-step reaction. *C. elegans* expresses a variety of UDP-glycosyltransferases which could catalyze the generation of an *O*-glucoside, followed by phosphorylation of the glucose moiety by the action of a hexokinase (**Figure 7**). 265 glycosyltransferase genes have been identified for *C. elegans* to date of which 29% belong to the UDP-glycosyltransferase family [19]. Preliminary experiments in our lab using RNA interference in *C. elegans* with the goal to identify UDP-glycosyltransferases that could catalyze the formation of the *O*-glucosides failed as in most cases silencing was lethal (data not shown). To identify the relevant enzymes involved in this pathway will be challenging.

Genistein-*O*-disaccharides and phosphorylated genistein-*O*-disaccharides were only formed in minor amounts. However, disaccharide conjugates are a new metabolite class as well and have never been reported as products of the *C. elegans* xenobiotic defense system. The pathway of their formation is equally unclear. We have not identified any hydroxylated genistein metabolites in worms known to be formed by cytochrome P450 enzymes in mammalian species (data not shown). However, even in mammals this reaction is only a side-pathway since genistein already possesses three hydroxyl groups.

**Figure 8:**
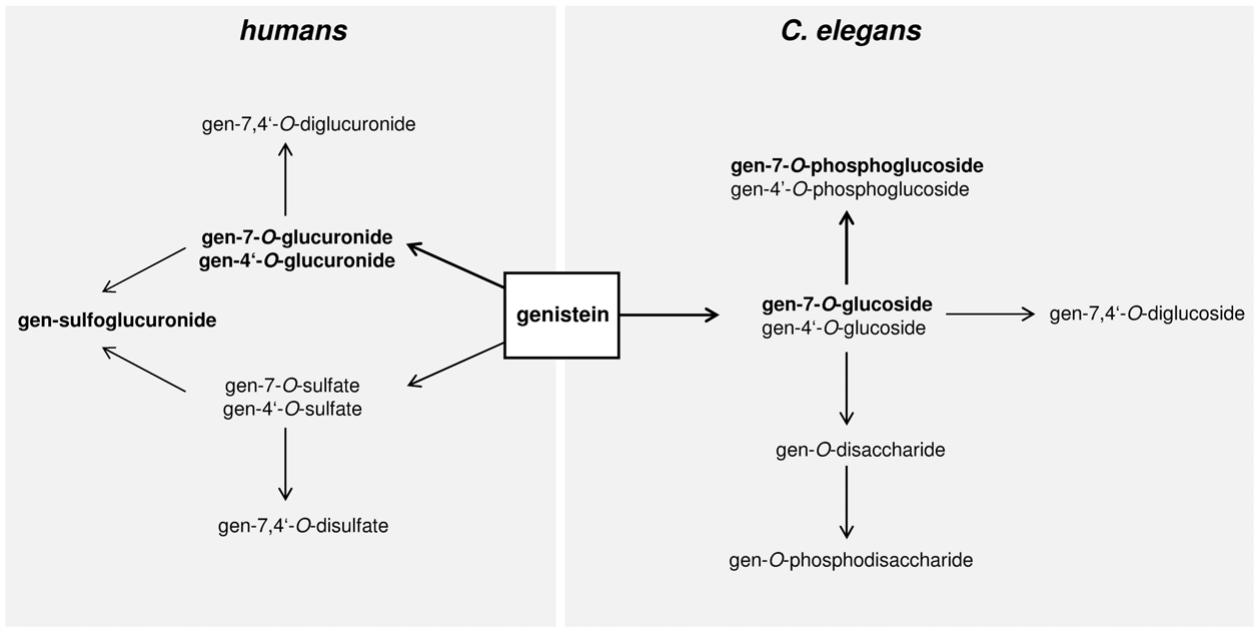
Main pathways for the conjugative metabolism of genistein in humans and *C. elegans*. Dominant metabolites are marked in bold.

**Figure 8** compares the conjugative metabolism of genistein in *C. elegans* and humans. In contrast to the formation of phosphoglucosides and glucosides described here for *C. elegans*, mammalian systems appear to metabolize genistein to glucuronides, sulfates and mixed sulfoglucuronides exclusively. [20] [21]. In addition, in humans, the microbial metabolism and degradation of genistein results in products like dihydrogenistein, 6′-hydroxy-*O*-desmethylangolensin, and 2-(4-hydroxyphenyl)-propionic acid which influence the bioactivity dramatically. In *C. elegans* none of these microbial metabolites were detected.

Very recently, Fischer et al. made the interesting observation that daidzein exhibits an estrogenic effect in *C. elegans* which is proposed to be responsible for lifespan prolongation of the nematode in the presence of pathogenic bacteria, whereas genistein acts in an antiestrogenic manner, diminishes the resistance against the pathogen and reduces lifespan under the same conditions [9]. Based on our results, we suggest that the effect observed might not be caused by the parent compound itself but by the phophoglucosides identified as the main metabolites that were formed. It is described that the nature of the moiety attached can affect the bioactivity of a metabolite considerably. Glucuronidation of daidzein at the 4′- and 7-position resulted, for example, in a significant reduction in estrogenicity, although the activity was not completely eliminated [22]. Sulfation of daidzein in the 4′-position only modestly influenced the estrogen agonist activity and even more, the 7-*O*-sulfate of daidzein exerted a much higher activity than daidzein itself [23]. In the case of genistein, the 7-*O*-sulfation acts in an opposite way and reduced the estrogenic activity substantially. However, not only estrogenicity has been shown to be affected by the type of conjugation. Daidzein sulfoconjugates are also described as potent inhibitors of sterol sulfatase while daidzein does not affect this enzyme [24].

In conclusion, our study provides evidence for a novel biotransformation pathway in *C. elegans* leading to the formation of glucoside- and phosphoglucosides derivatives which so far have not been identified in a mammalian system. The metabolism of genistein in mammals and *C. elegans* therefore shows huge differences which may greatly impact the bioactivity. These differences need to be appropriately taken into consideration when *C. elegans* is used as a model to assess possible health or aging effects.
